# Impact of Pre-Pregnancy BMI on B Vitamin and Inflammatory Status in Early Pregnancy: An Observational Cohort Study

**DOI:** 10.3390/nu8120776

**Published:** 2016-11-30

**Authors:** Anne-Lise Bjørke-Monsen, Arve Ulvik, Roy M. Nilsen, Øivind Midttun, Christine Roth, Per Magnus, Camilla Stoltenberg, Stein Emil Vollset, Ted Reichborn-Kjennerud, Per Magne Ueland

**Affiliations:** 1 Laboratory of Clinical Biochemistry, Haukeland University Hospital, 5021 Bergen, Norway; per.ueland@ikb.uib.no; 2 Bevital AS, 5021 Bergen, Norway; Arve.Ulvik@uib.no (A.U.); oivind.midttun@bevital.no (Ø.M.); 3 Centre for Clinical Research, Haukeland University Hospital, 5021 Bergen, Norway; Roy.Nilsen@uib.no; 4 The Norwegian Institute of Public Health, 0403 Oslo, Norway; Christine.Roth@fhi.no (C.R.); Per.Magnus@fhi.no (P.M.); camilla.stoltenberg@fhi.no (C.S.); SteinEmil.Vollset@fhi.no (S.E.V.); Ted.Reichborn-Kjennerud@fhi.no (T.R.-K.); 5 Nic Waals Institute, Lovisenberg Hospital, Oslo 0456, Norway; 6 Department of Public Health and Primary Health Care, University of Bergen, 5007 Bergen, Norway; 7 Institute of Clinical Medicine, University of Oslo, 0313 Oslo, Norway; 8 Department of Clinical Science, University of Bergen, 5007 Bergen, Norway

**Keywords:** pregnancy, obesity, pre-pregnancy BMI, B vitamins, inflammation

## Abstract

Maternal nutrition and inflammation have been suggested as mediators in the development of various adverse pregnancy outcomes associated with maternal obesity. We have investigated the relation between pre-pregnancy BMI, B vitamin status, and inflammatory markers in a group of healthy pregnant women. Cobalamin, folate, pyridoxal 5′-phosphate, and riboflavin; and the metabolic markers homocysteine, methylmalonic acid, and 3-hydroxykynurenine/xanthurenic acid ratio (HK/XA); and markers of cellular inflammation, neopterin and kynurenine/tryptophan ratio (KTR) were determined in pregnancy week 18 and related to pre-pregnancy body mass index (BMI), in 2797 women from the Norwegian Mother and Child Cohort Study (MoBa). Pre-pregnancy BMI was inversely related to folate, cobalamin, pyridoxal 5′-phosphate (PLP), and riboflavin (*p* < 0.001), and associated with increased neopterin and KTR levels (*p* < 0.001). Inflammation seemed to be an independent predictor of low vitamin B6 status, as verified by low PLP and high HK/XA ratio. A high pre-pregnancy BMI is a risk factor for low B vitamin status and increased cellular inflammation. As an optimal micronutrient status is vital for normal fetal development, the observed lower B vitamin levels may contribute to adverse pregnancy outcomes associated with maternal obesity and B vitamin status should be assessed in women with high BMI before they get pregnant.

## 1. Introduction

There is an increasing prevalence of obesity in most parts of the world, also affecting women of childbearing age [1]. Pre-pregnancy obesity is associated with an increased risk of adverse pregnancy outcomes for both mother and child, including subfertility, miscarriage, gestational diabetes, gestational hypertension, preeclampsia, macrosomia, preterm birth, congenital anomalies, and fetal death [2,3]. Epidemiological studies have revealed strong links between nutritional excess during pregnancy and later development of metabolic disease, such as type 2 diabetes and obesity, in adult life [4]. Maternal metabolic, nutritional, and inflammatory factors have been suggested as mediators in the development of the various negative pregnancy outcomes associated with maternal obesity.

A higher body mass index (BMI) has been associated with an adverse nutritional status in both non-pregnant adults [5,6,7] and children [8,9]. An inverse relation between pre-pregnancy BMI and several micronutrients like folate, vitamin D, carotenoids, zinc, and essential fatty acids have been reported in pregnant women, and negative pregnancy outcomes related to obesity might be related to impaired micronutrient status [10,11,12,13]. An adequate B vitamin status during pregnancy is important for maternal health and normal fetal development [14]. Deficiencies of folate, cobalamin, riboflavin, or vitamin B6 are associated with an increased risk of placental abruption, still-births, low birth weight, preterm deliveries, preeclampsia, as well as fetal malformations [14].

Obesity in itself results in an inflammatory state in metabolic tissues [15], and higher levels of the inflammatory markers C-reactive protein, neopterin, and kynurenine/tryptophan ratio (KTR) are seen in overweight and obese adults [16,17]. Inflammation has been associated with low circulating levels of several micronutrients [18], such as the active form of vitamin B_6_, pyridoxal 5′-phosphate (PLP) [19], and vitamin D [20].

The objective of the study was to investigate whether BMI and inflammation are independent determinants of B vitamin status, we investigated the association of pre-pregnancy BMI with plasma B vitamin status and inflammatory markers in pregnancy week 18 in 2797 women from the Norwegian Mother and Child Cohort Study (MoBa).

## 2. Experimental Section

### 2.1. Study Population

This study is based on a subsample of 2825 women included in the Norwegian Mother and Child Cohort Study (MoBa), a long-term, prospective study conducted by the Norwegian Institute of Public Health and including more than 100,000 Norwegian pregnant women and their infants during 1999–2008 [21]. The women included in this sub-study of MoBa gave a singleton birth between July 2002 and December 2003, and returned a baseline questionnaire including data on height and pre-pregnancy weight and donated a blood sample in pregnancy week 18, and were registered in the Medical Birth Registry of Norway. A detailed description of this sub population and the sampling procedures has been published [22].

### 2.2. Ethics

Written informed consent was obtained from each participant, and the study was approved by the Regional Committee for Medical Research Ethics Sør-Øst A, 19/10-2011 (permission number 2009/2593a) and the Norwegian Data Inspectorate. All questions used in the MoBa can be found online at www.fhi.no/morogbarn.

### 2.3. Blood Sampling and Laboratory Analyses

Maternal non-fasting blood samples were collected in ethylenediamine tetraacetic acid (EDTA) tubes in median pregnancy week 18. The samples were centrifuged within 30 min after collection and stored at 4 °C until shipped overnight to the Norwegian Institute of Public Health in Oslo. On the day of receipt (usually within 1–2 days), plasma was aliquoted into polypropylene microtiter plates and stored at −80 °C until analyses.

Plasma folate was determined by a *Lactobacillus casei* microbiological assay [23] and plasma cobalamin (vitamin B12) by a *Lactobacillus leichmannii* microbiological assay [24]. Concurrent intake of antibiotics may interfere with microbiological assays and cause falsely reduced plasma levels of the vitamins [25], and samples with plasma folate levels <2.33 nmol/L were excluded (*n* = 28, i.e., the lower one percentile), leaving a total of 2797 samples to be included in the study.

Plasma levels of total homocysteine (tHcy), a marker of folate and cobalamin status, and methylmalonic acid (MMA), a marker of cobalamin status, were assayed using a GC-MS method based on methylchloroformate derivatization [26]. Plasma levels of riboflavin (vitamin B2); pyridoxal 5’-phosphate (PLP; vitamin B6); the ratio between 3-hydroxykynurenine and xanthurenic acid (HK/XA), a marker of vitamin B6 status [27]; and two markers of interferon-gamma mediated cellular immune activation, neopterin [28] and KTR (Kynurenine/Tryptophan × 1000)—the latter reflecting indoleamine-2,3-dioxygenase (IDO) activation [29]—were analyzed using a liquid chromatography-tandem mass spectrometry assay [30]. All biomarkers were measured at the laboratory of Bevital AS (www.bevital.no).

### 2.4. Covariates

Data on maternal age at delivery, marital status, and parity were obtained from the Medical Birth Registry of Norway. Data on maternal education, smoking habits, pre-pregnancy BMI, weight increase to pregnancy week 18, and use of supplements were obtained from the self-reported baseline cohort questionnaire. Pre-pregnancy BMI (kg/m^2^) was coded as <18.5, 18.5–24.9, 25.0–29.9, 30.0–34.9, and ≥35.0. Use of supplements was coded as non-user, use of folic acid or other supplement, and use of folic acid plus other supplement, based on reported intake from four weeks prior to conception up to pregnancy week 18. A B vitamin summary score was calculated as a sum of quintiles for folate, cobalamin, PLP, and riboflavin.

### 2.5. Statistical Analyses

Values are presented as means with standard deviation (SD) or medians with the interquartile range. Differences between groups were examined by ANOVA, Mann-Whitney U test and the Kruskal Wallis test. Differences in categorical variables were assessed with the Chi-square test. Multiple-linear regression models were used to assess the relation between pre-pregnancy BMI, use of supplements, parity, age, and inflammation with plasma B vitamin status obtained around pregnancy week 18. The odds ratio (ORs) for having a low B vitamin status (quintile 1) according to maternal factors, including the inflammation marker neopterin, was assessed by logistic regression.

To explore non-linearity, we modeled pre-pregnancy BMI versus B vitamins and metabolic markers, using one-dimensional smoothing splines in Generalized Additive Models (GAM), in a model adjusted for adjusted for use of supplements, maternal age, parity, and neopterin.

GAMs were computed using the package mgcv (version 1.4–1) in R (The R Foundation for Statistical Computing, version 2.8.1, Vienna, Austria) [31], and the SPSS/PASW statistical program version 22 (IBM Corp, Armonk, NY, USA).the company, the city, the country) was used for all other statistical analyses. Two-sided *p*-values < 0.05 were considered statistically significant.

## 3. Results

### 3.1. Characteristics of the Study Population According to Pre-Pregnancy BMI

Demographic characteristics according to pre-pregnancy BMI are presented in Table 1. Fifty-eight percent of the women were multipara, and had a mean number of 1.5 (SD 0.7) former children, with no difference according to BMI group (*p* = 0.14). The majority of the women were healthy, a low percentage (0.7%) had chronic hypertension and 2% had had IVF treatment. The use of multivitamin supplements differed according to BMI group, with an increasing trend for not using any supplements with higher BMI (*p* < 0.001). Regular use of alcohol was rare and 96% reported drinking alcohol never or less than once a month after becoming pregnant. A total of 231 (8%) women reported daily smoking with a mean number of seven (SD five) cigarettes per day, with no significant difference in number between the smokers in the various BMI groups (*p* = 0.80). There was an inverse relation between pre-pregnancy BMI and weight gain to pregnancy week 18 (*r* = −0.24, *p* < 0.001) and to term (*r* = −0.16, *p* < 0.001).

Women in the highest pre-pregnancy BMI group (BMI ≥ 35) put on less weight during pregnancy, they were more likely to be multipara, not use micronutrient supplements, and to have a lower educational level (*p* < 0.01). The lowest mean birth weight was seen in women with a pre-pregnancy BMI <18.5 (Table 1).

### 3.2. Plasma B Vitamin Status According to Pre-Pregnancy BMI

**Figure 1:**
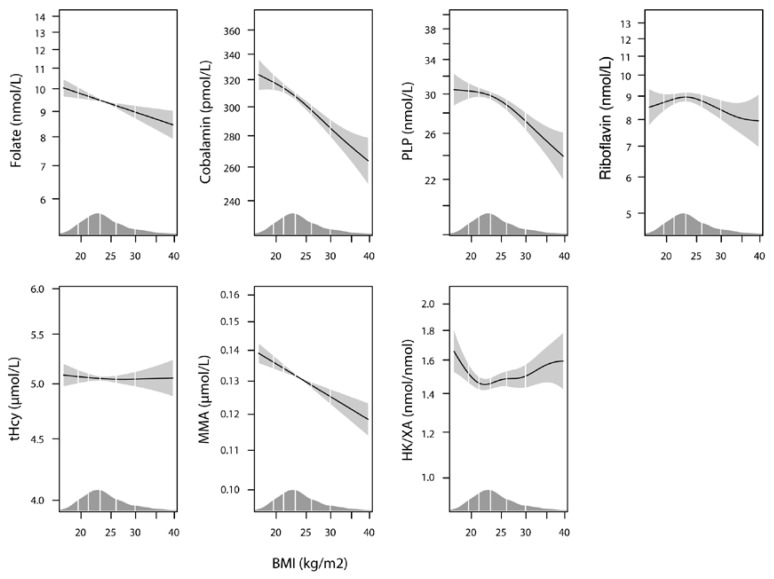
The association of pre-pregnancy BMI with cobalamin, folate, PLP, riboflavin, tHcy, MMA, and HK/XA by generalized additive models (GAM), adjusted for use of multisupplements, maternal age, parity, and neopterin. The solid line shows the fitted model and the shaded areas indicate 95% CIs. PLP, pyridoxal 5′phosphate; tHcy, total homocysteine; MMA, methylmalonic acid, HK/XA, 3-hydroxykynurenine/xanthurenic acid ratio.

There was a trend towards lower levels of B vitamins and higher levels of the metabolic markers with higher pre-pregnancy BMI (Table 2, Figure 1). The best B vitamin status was seen in women with a normal pre-pregnancy BMI (18.5–24.9), as they had the highest levels of folate, PLP, and riboflavin with the lowest tHcy and HK/XA ratio (Table 2). Cobalamin decreased with increasing BMI, but this was not reflected in the MMA levels, which were inversely related to pre-pregnancy BMI.

The circulating B-vitamins were inversely related to their functional markers, as expected. tHcy was inversely related to folate (*r* = −0.48, *p* < 0.001), less to cobalamin (*r* = −0.24, *p* < 0.001), PLP (*r* = −0.16, *p* < 0.001), and riboflavin (*r* = −0.15, *p* < 0.001). MMA was inversely related to cobalamin (*r* = −0.17, *p* < 0.001) and HK/XA to PLP (*r* = −0.29, *p* < 0.001). All B vitamins were positively related to each other (*r* > 0.12, *p* < 0.001).

### 3.3. Inflammation Markers According to Pre-Pregnancy BMI

**Figure 2:**
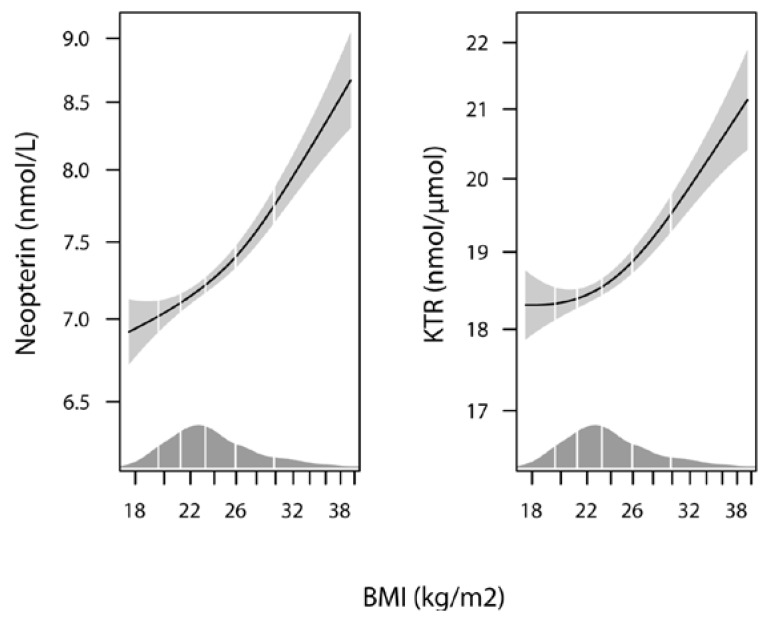
The association of pre-pregnancy BMI with neopterin and KTR by Generalized additive models (GAM), adjusted for use of multisupplements, maternal age, and parity. The solid line shows the fitted model and the shaded areas indicate 95% CIs. KTR, kynurenine/tryptophan ratio.

There was a positive correlation between pre-pregnancy BMI and the inflammation markers neopterin (*r* = 0.18, *p* < 0.001) and KTR (*r* = 0.13, *p* < 0.001), as demonstrated in Figure 2. Neopterin and KTR were strongly correlated to each other (*r* = 0.48, *p* < 0.001).

### 3.4. Maternal Factors and Inflammation Markers as Determinants of Plasma B Vitamin Status in Pregnancy Week 18

Use of supplements was the most consistent and strongest determinant for maternal B vitamin status, with significant influence on folate, cobalamin, PLP, riboflavin, tHcy, and HK/XA, in a multiple linear regression model, which additionally included pre-pregnancy BMI, age, parity, and neopterin (Table 3). In this model, pre-pregnancy BMI was a significant determinant of all vitamin markers except for riboflavin and tHcy, whereas parity was strongly negatively correlated to folate and PLP status. All parameters in the model were significantly correlated to the calculated B vitamin summary score (Table 3). Neopterin was significantly negative related to PLP, and positively to the metabolic markers tHcy, MMA, and HK/XA. Substituting neopterin with KTR in the model did not essentially change the results (data not shown). Including gestational weight gain in pregnancy week 18 as an additional factor did not change the results.

The OR for having a low calculated B vitamin summary score (quintile 1) was increased with pre-pregnancy BMI >30.0 and higher parity, and reduced with higher maternal age and use of supplements in a logistic regression model, which additionally included neopterin quartiles (Table 4). A high pre-pregnancy BMI was a predictor of low cobalamin and PLP levels, multiparity was a predictor of low folate levels, higher maternal age was associated with better folate and riboflavin status, whereas use of supplements was associated with better status of all four B vitamins. Increased inflammation, evaluated by higher neopterin levels, was only a predictor of low PLP in this model. Substituting neopterin with KTR in the model did not essentially change the results.

The relations between pre-pregnancy BMI and B vitamins and metabolic markers, visualized by GAM in a model which additionally corrected for use of supplements, maternal age, parity, and neopterin, are shown in Figure 1. The association curves for folate, cobalamin, PLP, and MMA show a linear negative relation to pre-pregnancy BMI, while the relation between pre-pregnancy BMI and the other biomarkers are inverse U-shaped (riboflavin), null (tHcy), or inverse in the lower range (HK/XA).

The association curves between pre-pregnancy BMI and the inflammation markers, neopterin and KTR, visualized by GAM in a model which also corrected for use of supplements, maternal age, and parity, show a strong linear relation through the whole range of BMI values (Figure 2).

## 4. Discussion

### 4.1. Main Findings

In this study of healthy women in pregnancy week 18, use of multi supplements was the strongest maternal determinant for B vitamin status. Pre-pregnancy BMI was inversely related to folate, cobalamin, PLP, and riboflavin levels, and associated with increased markers of cellular immune activation. Inflammation seemed to be an independent predictor of low PLP levels.

### 4.2. Strength and Limitations

All pregnant women in Norway were invited to participate in the MoBa study, but the attendance rate was only 38.5%, rendering the cohort not representative of the Norwegian population of pregnant women. The women who participated in the MoBa study are reported to be older, more frequently vitamin users, non-smokers, and primiparous [32]. Pre-pregnancy weight was self-reported around pregnancy week 18, and as obesity is an important determinant of underreporting BMI [33], self-reporting without verification is a limitation of the study.

The samples were stored at −80 °C before analyzing. The stability of plasma metabolites according to sample handling and storage conditions has been validated and the biomarkers investigated do not change significantly during storage at −80 °C [34].

C-reactive protein, CRP, an acute phase reactant and a commonly used inflammation marker, might have added additional information about low grade inflammation, but was not analyzed, which is a limitation of the study.

### 4.3. Interpretation

#### 4.3.1. Effects of Pre-Pregnancy BMI on Gestational Weight Gain and Birth Weight

In a former study of the MoBa cohort including approximately 58,000 mothers, the majority (65%) reported a normal pre-pregnancy BMI (18.5–24.9), whereas 2.9% were underweight (<18.5), and 2.6% were obese (≥35) [35], which is in accordance with the findings in the present substudy.

We observed a significant inverse relation between pre-pregnancy BMI and gestational weight gain up to pregnancy week 18 and throughout pregnancy, and despite this, a higher pre-pregnancy BMI was associated with a higher birth weight, which has been reported previously [35].

#### 4.3.2. Pregnancy Related Changes in Biochemical Parameters

Physiological changes in pregnancy, including hemodilution, altered renal function, hormonal status, and binding-protein concentrations, affect plasma B vitamin levels and the relation to their functional metabolic markers [36,37]. Folate, cobalamin, and PLP tend to decrease, whereas riboflavin is reported to show only minor changes during pregnancy [38,39,40,41,42]. tHcy, a marker of folate and cobalamin deficiency [43], is 30%–60% lower in pregnant compared to nonpregnant women, with the lowest levels seen in the second trimester [44]. MMA, a marker of cobalamin deficiency [45], is reported to be reduced during the first two trimesters, thereafter gradually increasing [46]. We observed a strong inverse relation between folate and tHcy, and a weaker relation between cobalamin and MMA, as observed by others [36,47,48].

The ratio HK/XA has been shown to have a strong negative correlation with plasma levels of PLP, the active form of vitamin B6, and has been proposed as a potential markers of functional vitamin B6 status [27]. The inverse relation between PLP and HK/XA has not been previously documented in pregnant women, but we observed lower PLP and higher HK/XA ratios with increasing pre-pregnancy BMI, indicating a functional vitamin B6 deficiency, particularly in women with a pre-pregnancy BMI ≥30.

Extensive changes in the maternal immune system are necessary for maintaining a normal pregnancy [49] and both neopterin levels and KTR are reported to increase during pregnancy [50] Neopterin levels correlate with gestational age (*r* = 0.28, *p* = 0.001) and are reported to be higher in pregnancies complicated with preterm birth and preeclampsia [51,52,53].

#### 4.3.3. Maternal Predictors for B Vitamin Status

An inverse relation between pre-pregnancy BMI and folate, vitamin D, carotenoids has formerly been reported [10,11,13]. Our results have expanded this knowledge by reporting also lower levels of PLP (vitamin B6), riboflavin (vitamin B2), and cobalamin (vitamin B12) with higher pre-pregnancy BMI.

Pre-pregnancy BMI, use of multisupplements, parity, and maternal age were all related to B vitamin status. We observed a slightly U-shaped relation between pre-pregnancy BMI and B vitamin levels in pregnancy week 18, with the best status seen in women with a normal pre-pregnancy BMI (18.5–24.9), slightly lower in underweight women (BMI < 18.5) and lowest in obese women (BMI ≥35.0). The strongest maternal determinant for B vitamin status was use of multisupplements, and the percentage of users was higher in normal weight compared to obese women, which might explain the inverse relation between pre-pregnancy BMI and vitamin status. Use of micronutrient supplements during pregnancy has been associated with better socioeconomic status [48], known to be related to a better diet, maternal health, and pregnancy outcome [54]. Multiparity (≥3) was associated with an increased risk of folate deficiency, while maternal age >25 years was associated with a better folate status. In the MoBa cohort, the 10% who had used supplements regularly from one month before pregnancy throughout the first trimester (as recommended) were more likely to be older, married, nonsmokers, with a higher income and lower parity [54]. Compared to women with a lower pre-pregnancy BMI, obese women (BMI ≥ 35) had not increased their weight in pregnancy week 18, when the blood samples were collected, which might contribute to their lower micronutrient status.

#### 4.3.4. The Complex Relation between Pre-pregnancy BMI, Inflammation, and Vitamin Levels

We observed a linear relation between pre-pregnancy BMI and the markers of cellular immune activation, neopterin and KTR, as previously reported in non-pregnant adults [16]. Inflammation is reported to be associated with lower plasma levels of several micronutrients, particularly vitamin B6 and vitamin D [18,19,20], but the magnitude of effect varies between different micronutrients and individuals [18,55,56]. The lower PLP levels associated with inflammation have been proposed to be due to increased PLP degradation [57] and to PLP redistribution from plasma to tissues [58], and PLP have been suggested to reflect interferon-γ-mediated immune activation [59].

In this study, neopterin was inversely related to PLP and positively to HK/XA, but not to any other vitamin, in both multiple linear and logistic regression models, which included pre-pregnancy BMI and other maternal factors. The increase in the functional marker, HK/XA, suggests that low PLP does not merely refelect altered distribution, but that inflammation may be an independent risk factor for lower vitamin B6 status.

## 5. Conclusions

A high pre-pregnancy BMI is associated with lower levels of folate, cobalamin, PLP, and riboflavin; and increased levels of the cellular inflammation markers, neopterin, and KTR. As an optimal micronutrient status is vital for normal fetal development, the observed lower B vitamin levels may contribute to the negative pregnancy outcomes associated with maternal obesity. Assessment of B vitamin status should be encouraged in women with a high pre-pregnancy BMI when planning a pregnancy.

## Figures and Tables

**Table 1:** Maternal characteristics according to maternal pre-pregnancy BMI, *n* = 2797.

	Pre-pregnancy BMI, Categories					*p* Value
	<18.5	18.5–24.9	25.0–29.9	30.0–34.9	≥35.0	
	*n* = 80 (3%)	*n* = 1827 (65%)	*n* = 587 (21%)	*n* = 213 (8%)	*n* = 90 (3%)	
Primipara, *n* (%)	47 (59%)	785 (43%)	231 (39%)	86 (40%)	26 (29%)	0.001 ^b^
Age, years, mean (SD)	27.8 (4.9)	29.9 (4.5)	29.9 (4.6)	29.8 (4.6)	29.5 (4.8)	0.003 ^a^
Education						
Primary school, *n* (%)	9 (12%)	39 (2%)	15 (3%)	11 (5%)	6 (7%)	<0.001 ^b^
Secondary school, *n* (%)	30 (41%)	623 (36%)	273 (48%)	97 (47%)	48 (55%)	
University or college, *n* (%)	35 (47%)	1077 (62%)	286 (50%)	98 (48%)	34 (39%)	
Use of supplements anytime from four weeks before pregnancy up to pregnancy week 18	63 (79%)	1523 (83%)	470 (79%)	156 (72%)	61 (68%)	<0.001 ^b^
Use of alcohol (≥1/month), *n* (%)	5 (0.2%)	83 (3%)	15 (0.6%)	1 (0%)	3 (0.1%)	0.009 ^b^
Daily smoking, *n* (%)	13 (16%)	140 (8%)	47 (8%)	22 (10%)	9 (10%)	0.04 ^b^
Weight increase, kg, mean (SD), (% of pre-pregnancy weight)						
to pregnancy week 18	4.5 (3.1) (9%)	3.3 (2.9) (5%)	2.4 (3.2) (3%)	1.1 (3.5) (1%)	−0.1 (4.3) (−0.1%)	<0.001 ^a^ <0.001 ^a^
to birth	17.3 (6.3) (35%)	15.5 (5.9) (25%)	14.8 (6.7) (20%)	11.5 (7.3) (13%)	8.7 (8.9) (8%)	<0.001 ^a^ <0.001 ^a^
Birth weight, g, mean (SD)	3343 (635)	3575 (588)	3727 (600)	3696 (648)	3758 (755)	<0.001 ^a^

^a^ ANOVA: A one-way variance analysis; ^b^ Pearson’s Chi Square test. BMI: body mass index; SD: the standard deviation.

**Table 2:** Maternal plasma levels of vitamins, metabolic and inflammation markers in pregnancy week 18 according to pre-pregnancy BMI, *n* = 2797.

	Pre-pregnancy BMI; Categories					*p* Value
	<18.5	18.5–24.9	25.0–29.9	30.0–34.9	≥35.0	
	*n* = 80	*n* = 1827	*n* = 587	*n* = 213	*n* = 90	
Plasma folate, nmol/L ^a^	8.8 (6.0–15.7)	9.2 (6.3–16.0)	8.1 (5.7–12.8)	7.3 (5.1–12.2)	7.3 (5.0–12.1)	<0.001
Plasma cobalamin, pmol/L ^a^	328 (243–383)	314 (254–386)	301 (240–363)	271 (220–338)	268 (204–317)	<0.001
Plasma PLP, nmol/L ^a^	27.0 (21.4–39.7)	28.3 (21.8–40.7)	26.6 (20.6–35.6)	23.8 (17.4–33.7)	21.5 (17.0–30.1)	<0.001
Plasma riboflavin, nmol/L ^a^	7.8 (4.8–14.4)	8.1 (5.6–13.2)	7.8 (5.2–13.9)	7.2 (5.2–11.0)	6.9 (4.4–10.1)	0.004
B vitamin status ^a,b^	12.2 (3.7)	12.5 (3.6)	11.7 (3.7)	10.6 (3.7)	10.1 (3.4)	<0.001
Plasma tHcy, µmol/L ^a^	5.00 (4.22–5.92)	4.91 (4.27–5.79)	4.94 (4.23–5.78)	5.04 (4.44–6.21)	5.33 (4.43–6.13)	0.03
Plasma MMA, µmol/L ^a^	0.13 (0.11–0.16)	0.13 (0.11–0.16)	0.13 (0.10–0.16)	0.12 (0.10–0.15)	0.12 (0.10–0.15)	0.009
Plasma HK/XA ^a^	1.6 (1.2–2.1)	1.4 (1.0–2.0)	1.5 (1.1–2.1)	1.7 (1.2–2.2)	1.8 (1.2–2.4)	<0.001
Plasma neopterine, µmol/L ^a^	6.7 (6.1–8.1)	7.0 (6.1–8.1)	7.3 (6.3–8.6)	7.9 (7.0–9.1)	8.5 (7.31–9.9)	<0.001
KTR, nmol/µmol ^a^	18.1 (16.0–21.5)	18.3 (16.2–20.6)	18.8 (16.8–21.2)	19.6 (17.5–22.8)	21.2 (18.5–24.0)	<0.001

^a^ Median (IQR), by Kruskal Wallis test; ^b^ B vitamin status; summary score based on added quintiles of the 4 B vitamins, Mean (SD), by ANOVA; tHcy, total homocysteine; MMA, methylmalonic acid; HK/XA: 3-hydroxykynurenine/xanthurenic acid; KTR, kynurenine/tryptophan ratio.

**Table 3:** Determinants of maternal plasma B vitamin status in pregnancy week 18 by multiple linear regression, *n* = 2797.

	Plasma Folate, nmol/L	Plasma Cobalamin, pmol/L	Plasma PLP, nmol/L	Plasma Riboflavin, nmol/L	Plasma tHcy, µmol/L	Plasma MMA, µmol/L	Plasma HK/XA	B Vitamin Score ^a^
	B (95% CI)	B (95% CI)	B (95% CI)	B (95% CI)	B (95% CI)	B (95% CI)	B (95% CI)	B (95% CI)
Pre-pregnancy BMI ^c^	−0.59 ** (−0.98, −0.20)	−16.7 ** (−22.0, −11.4)	−2.55 ** (−3.72, −1.38)	−0.60 (−1.25, 0.06)	−0.02 (−0.16, 0.12)	−0.006 ** (−0.008, −0.003)	0.11 * (0.04, 0.18)	−0.47 ** (−0.60, −0.35)
Parity ^e^	−1.33 ** (−1.72, −0.95)	−3.6 (−8.8, 1.6)	−1.28 * (−2.43, −0.12)	−1.08 * (−1.73, −0.43)	−0.01 (−0.15, 0.13)	−0.001 (−0.004, 0.002)	−0.03 (−0.09, 0.04)	−0.34 ** (−0.47, −0.22)
Age ^d^	2.69 ** (2.05, 3.32)	12.3 * (3.6, 20.9)	1.96 * (0.06, 3.86)	0.30 (−0.77, 1.36)	0.04 (−0.19, 0.27)	0.002 (−0.002, 0.007)	−0.12 * (−0.24, −0.01)	0.63 ** (0.43, 0.83)
Use of supplements ^b^	5.14 ** (4.35, 5.94)	13.0 * (2.3, 23.7)	8.49 ** (6.12, 10.85)	1.80 * (0.48, 3.13)	−0.75 ** (−1.03, −0.47)	0.001 (−0.004, 0.007)	−0.21 * (−0.35, −0.07)	1.73 ** (1.48, 1.98)
Neopterin ^f^	−0.12 (−0.35, 0.11)	−1.9 (−5.0, 1.2)	−1.00 * (−1.68, −0.31)	−0.08 (−0.47, 0.30)	0.10 * (0.02, 0.18)	0.003 ** (0.001, 0.004)	0.19 ** (0.15, 0.23)	−0.12 ** (−0.20, −0.05)

All variables were included in the model; ^a^ B vitamin score; added quintiles of folate, cobalamin, PLP and riboflavin; ^b^ No use versus use of supplements any time from four weeks before pregnancy up to pregnancy week 18; ^c^ Pre-pregnancy BMI, categorized; <18.5, 18.5-24.9, 25.0-29.9, 30.0-34.9, ≥35.0; ^d^ Age, categorized; <25 years, 25–35 years, >35 years; ^e^ Parity, categorized; 0, 1, 2, 3+; ^f^ Neopterin, quintiles; ≤5.84, 5.85–6.68, 6.69–7.50, 7.51–8.67, ≥8.68;* *p* value < 0.05; ** *p* value < 0.001. CI: indicates confidence interval.

**Table 4:** OR for low B vitamin status according to maternal factors, by logistic regression (*n* = 2797).

Independent Variables	OR (95% CI) for				
	Plasma Folate <5.45 nmol/L (Quintile 1)	Plasma Cobalamin <230 pmol/L (Quintile 1)	Plasma PLP <19.26 µmol/L (Quintile 1)	Plasma Riboflavin <4.91 µmol/L (Quintile 1)	B Vitamin Score ^a^ (Quintile 1)
Pre-pregnancy BMI (vs. category 2 18.5–24.9)					
<18.5	1.0 (0.5–1.8)	1.1 (0.6–1.9)	1.0 (0.5–1.8)	1.7 (1.0–2.8)	1.1 (0.7–1.9)
25.0–29.9	1.1 (0.9–1.5)	1.3 (1.0–1.6)	1.3 (1.0–1.6)	1.3 (1.0–1.7)	1.5 (1.2–1.8)
30.0–34.9	1.5 (1.0–2.1)	2.1 (1.5–2.9)	2.1 (1.6–3.0)	1.4 (1.0–2.0)	2.7 (2.0–3.7)
≥35.0	1.7 (1.0–2.8)	2.3 (1.4–3.6)	2.2 (1.4–3.5)	1.8 (1.1–3.0)	2.8 (1.7–4.3)
*p* trend	0.006	<0.001	<0.001	0.001	<0.001
Parity (vs. primipara)					
Para 1	1.2 (1.0–1.6)	1.1 (0.9–1.4)	1.3 (1.0–1.6)	1.3 (1.0–1.6)	1.3 (1.0–1.6)
Para 2	1.7 (1.2–2.3)	1.3 (1.0–1.7)	1.1 (0.8–1.5)	1.2 (0.9–1.6)	1.6 (1.2–2.1)
Para 3+	3.2 (2.1–4.9)	1.6 (1.0–2.4)	1.1 (0.7–1.8)	1.4 (0.9–2.2)	2.1 (1.4–3.1)
*p* trend	<0.001	0.02	0.36	0.11	<0.001
Maternal age (vs. ≤25 years)					
25–35	0.4 (0.3–0.6)	0.7 (0.6–1.0)	0.7 (0.7–1.0)	0.7 (0.6–1.0)	0.5 (0.4–0.7)
≥35	0.3 (0.2–0.5)	0.8 (0.5–1.2)	0.8 (0.5–1.1)	0.6 (0.4–0.9)	0.4 (0.3–0.6)
*p* trend	<0.001	0.11	0.11	0.004	<0.001
Use of supplements (vs. non-user)					
User	0.2 (0.2–0.2)	0.8 (0.6–1.0)	0.5 (0.4–0.6)	0.6 (0.5–0.8)	0.3 (0.3–0.4)
*p* trend	<0.001	0.03	<0.001	<0.001	<0.001
Neopterin (vs. ≤6.20 µmol/L, Quartile 1)					
6.21–7.17	1.1 (0.8–1.4)	1.1 (0.8–1.5)	1.1 (0.8–1.5)	0.9 (0.7–1.2)	1.0 (0.8–1.3)
7.18–8.40	1.3 (1.0–1.8)	1.0 (0.8–1.4)	1.1 (0.8–1.4)	0.9 (0.7–1.1)	1.1 (0.9–1.4)
≥8.41	1.2 (0.9–1.6)	1.2 (0.9–1.6)	1.7 (1.3–2.2)	0.9 (0.7–1.2)	1.2 (1.0–1.6)
*p* trend	0.11	0.24	<0.001	0.38	0.05

All variables were included in the model; ^a^ B vitamin score; added quintiles of folate, cobalamin, PLP and riboflavin. OR: Odds ratio.

## Abbreviations

MoBa: the Norwegian Mother and Child Cohort; tHcy: total homocysteine; MMA: methylmalonic acid; HK: 3-hydroxykynurenine; XA: xanthurenic acid; PLP: pyridoxal 5’-phosphate; KTR: kynurenine/tryptophan ratio; IDO: indoleamine-2,3-dioxygenase.
